# Potential Effects of Ibuprofen, Remdesivir and Omeprazole on Dexamethasone Metabolism in Control Sprague Dawley Male Rat Liver Microsomes (Drugs Often Used Together Alongside COVID-19 Treatment)

**DOI:** 10.3390/molecules27072238

**Published:** 2022-03-30

**Authors:** Amira Hussain, Declan P. Naughton, James Barker

**Affiliations:** School of Life Sciences, Pharmacy and Chemistry, Kingston University, Kingston-upon-Thames, London KT1 2EE, UK; d.naughton@kingston.ac.uk (D.P.N.); j.barker@kingston.ac.uk (J.B.)

**Keywords:** cytochrome P450, remdesivir, omeprazole, ibuprofen, rat liver microsomes, CYP3A activity

## Abstract

The role of individual cytochrome P450 (CYPs) responsible for the drug metabolism can be determined through their chemical inhibition. During the pandemic, dexamethasone and remdesivir with omeprazole were used for the treatment of COVID-19, while Ibuprofen was taken to treat the symptoms of fever and headache. This study aimed to examine the potency of ibuprofen remdesivir, and omeprazole as inhibitors of cytochrome P450s using rat liver microsomes in vitro. Dexamethasone a corticosteroid, sometimes used to reduce the body’s immune response in the treatment of COVID-19, was used as a probe substrate and the three inhibitors were added to the incubation system at different concentrations and analysed by a validated High Performance Liquid Chromatography (HPLC) method. The CYP3A2 isoenzyme is responsible for dexamethasone metabolism in vitro. The results showed that ibuprofen acts as a non-competitive inhibitor for CYP3A2 activity with K_i_ = 224.981 ± 1.854 µM and IC_50_ = 230.552 ± 2.020 µM, although remdesivir showed a mixed inhibition pattern with a K_i_ = 22.504 ± 0.008 µM and IC_50_ = 45.007 ± 0.016 µM. Additionally, omeprazole uncompetitively inhibits dexamethasone metabolism by the CYP3A2 enzyme activity with a K_i_ = 39.175 ± 0.230 µM and IC_50_ = 78.351 ± 0.460 µM. These results suggest that the tested inhibitors would not exert a significant effect on the CYP3A2 isoenzyme responsible for the co-administered dexamethasone drug’s metabolism in vivo.

## 1. Introduction

The drug-metabolizing enzyme system, also known as the CYP450s superfamily, is responsible for the biotransformation of a large number of endogenous (fatty acids, hormones, bile acids, steroids, prostaglandins) and exogenous compounds (toxic chemicals, carcinogens, drugs, organic solvents, environmental pollutants) [1]. The use of a combination of medicine can result in potential drug–drug interactions, which, in turn, can change the drug metabolism in both Phase I and Phase II [2]. Many drug–drug interactions change the pharmacokinetic behaviour of the drugs in addition to the drug’s bioavailability (absorption, distribution, and elimination) [2]. In the majority of cases, the detoxification of the substrate occurs due to the action of CYP450, either as a direct effect or through the phase II enzyme’s actions. To date, biotransformation of most of the drugs is catalysed by CYP1, CYP2, and CYP3 families in the clinic [3]. The CYP3A isoenzymes metabolize almost 50% of clinical therapeutic drugs [4].

CYP3A4 is primarily accountable for 6β-hydroxylation of testosterone, cortisol, progesterone, and androstenedione [5]. CYP3A2 isoform in male rats is the major contributor to testosterone 6β-hydroxylation [6]. Shayeganpou et al. validated the role of rat CYP3A2 isoform in the metabolism of amiodarone, while CYP3A4 metabolises amiodarone in the human liver [7]. In another in vitro study, the effects of plumbagin on CYP3A2/4 activities, both in rat and human liver microsomes, were investigated [8]. Previous studies have shown that CYP3A4 is accountable for dexamethasone metabolism and that dexamethasone CYP3A4 substrate can competitively inhibit other drugs which are strong substrates for CYP3A4 [9]. CYP3A2 is abundantly expressed in rat liver microsomes and metabolises several drugs of clinical importance [10]. The CYP3A2 isoform in male-specific rat liver microsomes (RLMs) is accountable for 6-hydroxylation of dexamethasone (corticosteroid) and is close to the human metabolite profile [11]. Li et al. have reported that dexamethasone is a significant inducer of both rat CYP3A1/2 and human CYP3A4 [12].

**Figure 1:**
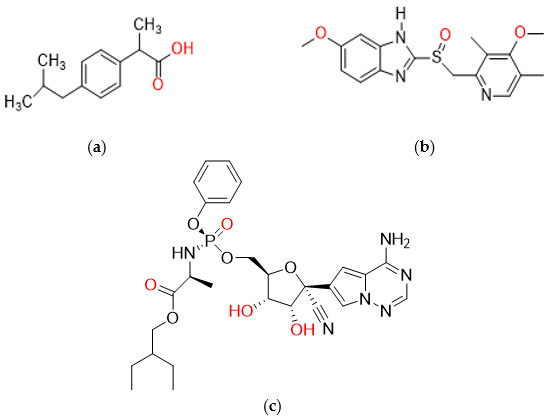
Chemical structures illustrating: (**a**) ibuprofen; (**b**) omeprazole; (**c**) remdesivir.

COVID-19 is a mainly self-limited disease and up to 20% of the cases will develop severe symptoms such as hypercoagulation, acute respiratory distress syndrome, pneumonia, and multiorgan system dysfunction. COVID-19 infection has resulted in >1.2 million deaths among 47 million recorded cases [13]. Various drugs against SARS-CoV-2 have been investigated and have been used to reduce the mortality caused by COVID-19, such as remdesivir, famotidine, and omeprazole (Figure 1) [14]. In a clinical study, dexamethasone appeared to reduce the death rate by 35% in ventilated intensive care unit (ICU) patients and 20% in nonventilated patients with supplemental oxygen [15]. Remdesivir is an antiviral drug that was originally used to treat hepatitis C, while omeprazole is a proton pump inhibitor and is used to treat excessive gastric acid production in the body. A reduction in recovery time in ICU patients has been associated with remdesivir therapy [15]. Aguila and Cua indicated in their study that remdesivir with omeprazole may represent therapeutic candidates for the treatment of COVID-19 [14]. Ibuprofen is a nonsteroidal anti-inflammatory drug and is used to treat inflammation, fever, and pain. It has been reported in a study that ibuprofen related mortality rates were lower compared to laxative-related mortality [13]. It was found in a randomized trial in the UK that ibuprofen helps to decrease the infection severity of acute respiratory tract infection in patients [16].

There are no data relating to the effects of ibuprofen, remdesivir, and omeprazole on dexamethasone metabolism (CYP3A2 activity). Our previous study showed that aspirin exhibits a weak competitive inhibition of CYP3A2 isoenzyme activity and has little potential to cause drug–drug toxicity [17].

Thus, in this report, we aimed to study the potential inhibitory impacts of these three compounds on the activity of CYP3A2 isoenzymes in rat liver microsomes using dexamethasone as a probe substrate in the presence of different concentrations of inhibitor drugs. These findings could provide significant information for the safe co-administration of these drugs in clinical settings.

## 2. Results and Discussion

### 2.1. HPLC Method Development and Validation

**Figure 2:**
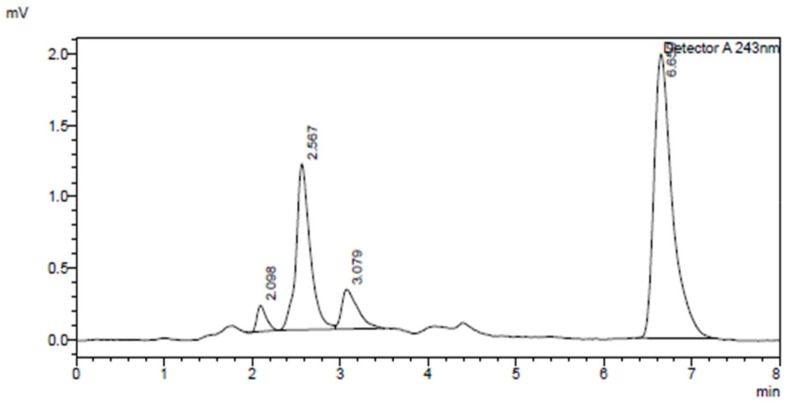
HPLC chromatogram displaying CYP3A2 assay components: ibuprofen (tR: 2.098), 6β-hydroxydexamethasone (tR: 2.567), dexamethasone (tR: 3.079) and 4−hydroxyoctanophenone (IS) (tR: 6.650) separation.

The HPLC method was developed and validated according to the ICH guidelines in terms of specificity, linearity, accuracy, and precision. Separation of dexamethasone, 6β-hydroxydexamethasone, inhibitors (ibuprofen, remdesivir, and omeprazole), and internal standard were attained using conditions presented in Table 1. The method showed specificity with metabolite (6β-hydroxydexamethasone) separated from other compounds with sufficient resolution. A representative chromatogram is shown in Figure 2.

**Figure 3:**
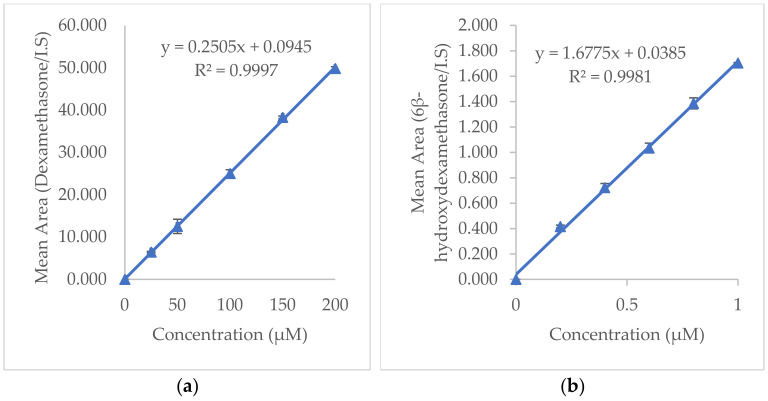
Calibration curves displaying linearity over the selected range: (**a**) dexamethasone; (**b**) 6β−hydroxydexamethasone.

The obtained calibration curves for dexamethasone and 6β-hydroxydexamethasone (Figure 3) showed good linearity over the concentration range of 25–200 µM and 0.2–1 µM, respectively. The representative linear equation for dexamethasone was y = 0.2505x + 0.0945 and for 6β-hydroxydexamethasone was y = 1.6775x + 0.0385 with a correlation coefficient (r^2^) of 0.99. For dexamethasone, good intra-sample and inter-sample precision were achieved with %RSD (% relative standard deviation) < 5%.

Good intra–day and inter-day precision was also obtained with %RSD < 10% for 6β-hydroxydexamethasone. The standard deviation ranged between 0.01% and 3.13%. The percentage recovery values for dexamethasone were between 81.68% and 112.52% and for 6β-hydroxydexamethasone between 93.13% and 119.38% (ICH acceptance criteria: %Recovery: 80–120%), representing good accuracy.

The developed method could be optimally performed with slight variations in peak areas, retention time, and peak heights. The calculated LOD and LOQ for dexamethasone were 5.60 µM and 16.98 µM, respectively. LOD and LOQ for 6β-hydroxydexamethasone were calculated to be 0.06 µM and 0.19 µM, respectively.

### 2.2. Optimisation of Substrate Concentration for Incubation System In Vitro

**Figure 4:**
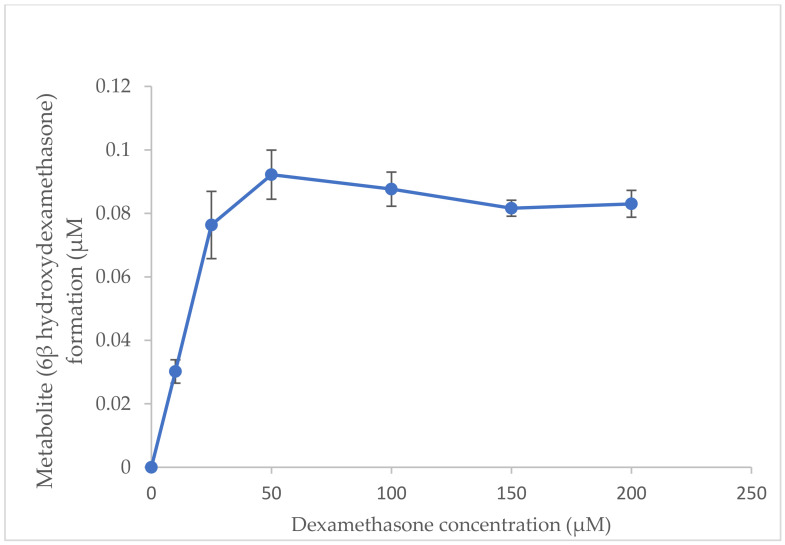
Effects of substrate concentration on 6β−hydroxydexamethasone formation.

The optimised incubation time was 40 min [17]. For optimisation of substrate concentration, a series of dexamethasone concentrations (10–200 µM) was added to the incubation system in vitro. The formation rate of the metabolite (6β-hydroxydexamethasone) from the substrate (dexamethasone) was increased up to 50 µM and then it became linear (Figure 4). Thus, the optimal substrate concentration range for the CYP3A2 inhibition study was 10–50 µM.

### 2.3. Inhibitory Effects of Ibuprofen on CYP3A2 Enzyme Activity in Rat Liver Microsomes (RLMs)

**Figure 5:**
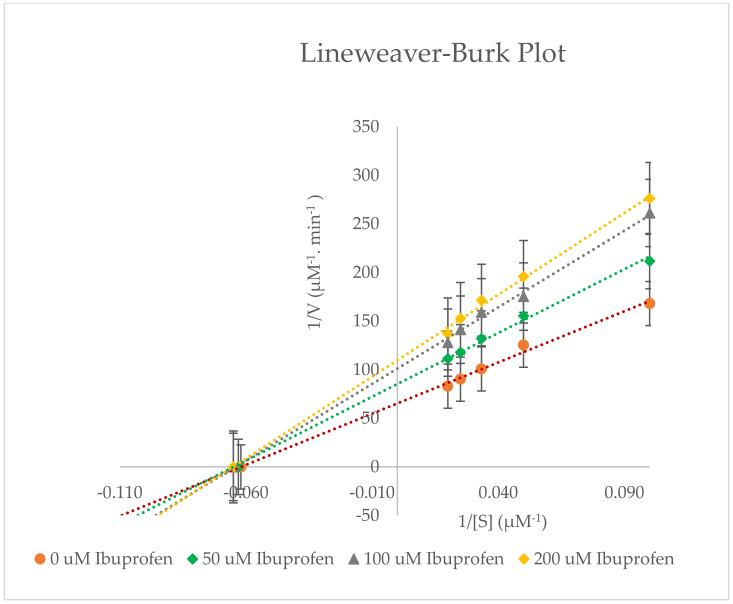
Lineweaver−Burk plot for the inhibition of CYP3A2−mediated dexamethasone metabolism by various concentrations of ibuprofen (0−200 μM) in rat liver microsomes. Values are expressed as the average of triplicate measurements.

**Figure 6:**
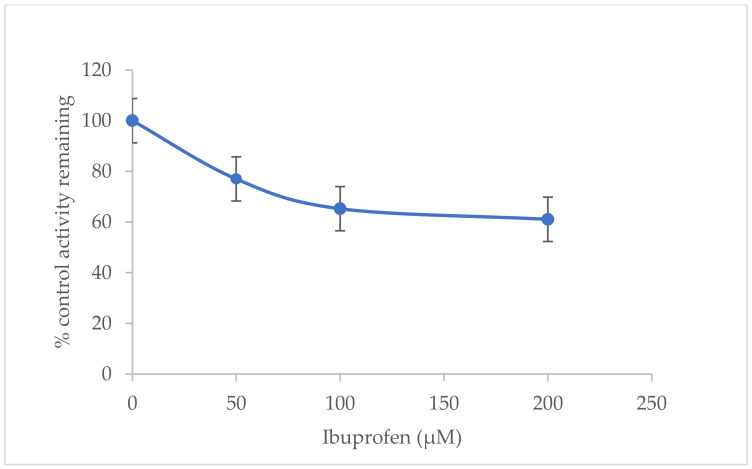
Inhibition of CYP3A2 enzyme activity by ibuprofen (0−200 µM) in rat liver microsomes. Values are expressed as the average of triplicate measurements. Control activity was taken as 100%.

Rat liver microsomes (0.5 mg/mL) were incubated with various concentrations of dexamethasone (10–50 µM) and ibuprofen (0–100 µM) in the presence of a NADPH regenerating system for 40 min at 37 °C. The metabolite was extracted using ethyl acetate and diethyl ether. The rates of 6β-hydroxydexamethasone formation were determined using High-Performance Liquid Chromatography (HPLC) technique. Figure 5 and Figure 6, and Table 2 show the inhibition of CYP3A2 activity by ibuprofen with apparent K_m_, V_max_, % inhibition, and Cl_int_ values (±SD).

### 2.4. Inhibitory Effects of Remdesivir on CYP3A2 Enzyme Activity in RLMs

**Figure 7:**
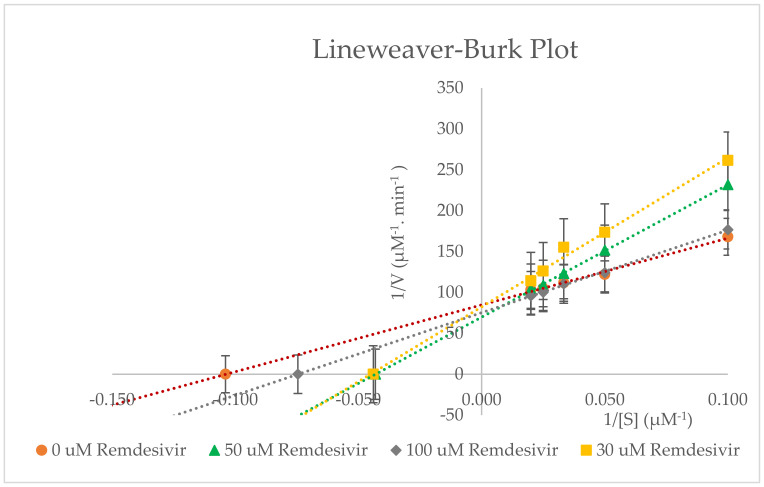
Lineweaver−Burk plot for the inhibition of CYP3A2−mediated dexamethasone metabolism by various concentrations of remdesivir (0−100 μM) in rat liver microsomes. Values are expressed as the average of triplicate measurements.

**Figure 8:**
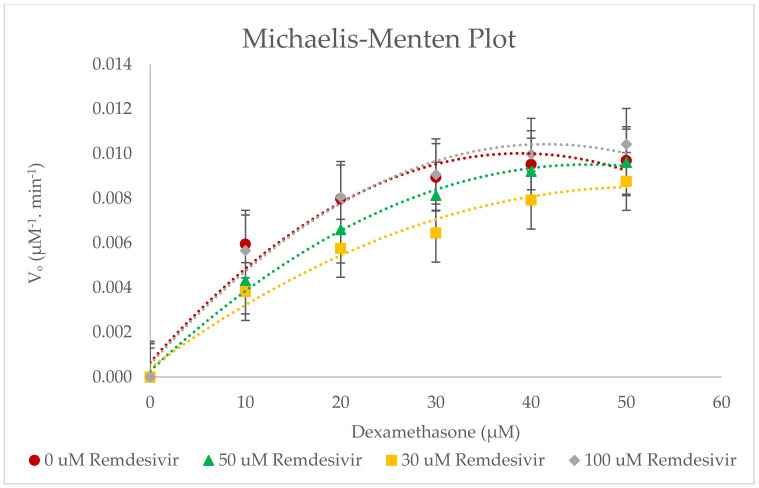
Michaelis−Menten plot for the inhibition of CYP3A2−catalysed dexamethasone 6−hydroxylation by remdesivir (0−100 µM). The polynomial function of order 2 was used to fit the curves. Values are expressed as the average of triplicate measurements.

Incubation of probe substrate dexamethasone (10–50 µM) with multiple remdesivir concentrations (0–100 µM) in RLMs showed that remdesivir inhibits CYP3A2 activity as a mixed inhibitor (Figure 7 and Figure 8). These primary data were then utilised to construct Lineweaver–Burk and Michaelis–Menten plots for the inhibition of dexamethasone metabolism (CYP3A2 activity) by remdesivir in RLMs. The obtained experimental data were analysed in triplicate and applied to estimate the resultant K_i_ (inhibition constant) and IC_50_ (half-maximum inhibitory concentration) values. Table 3 summarises the mean K_m_, V_max_, Cl_int_, IC_50_, and K_i_ values (±SD).

### 2.5. Inhibitory Effects of Omeprazole on CYP3A2 Enzyme Activity in RLMs

**Figure 9:**
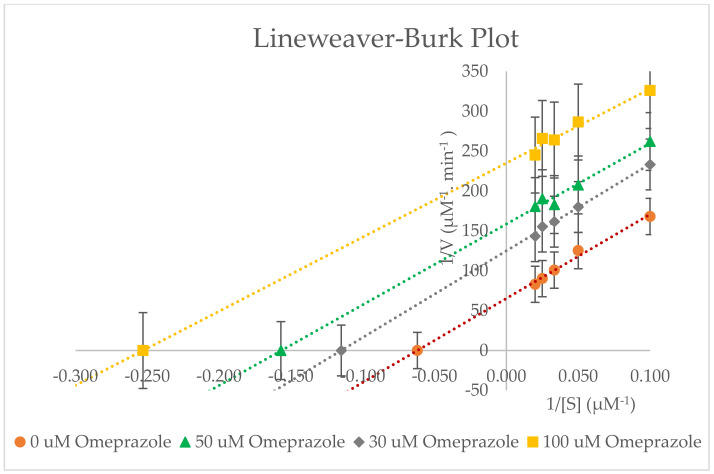
Lineweaver−Burk plot for the inhibition of CYP3A2−mediated dexamethasone metabolism by various concentrations of omeprazole (0−100 μM) in rat liver microsomes. Values are expressed as the average of triplicate measurements.

**Figure 10:**
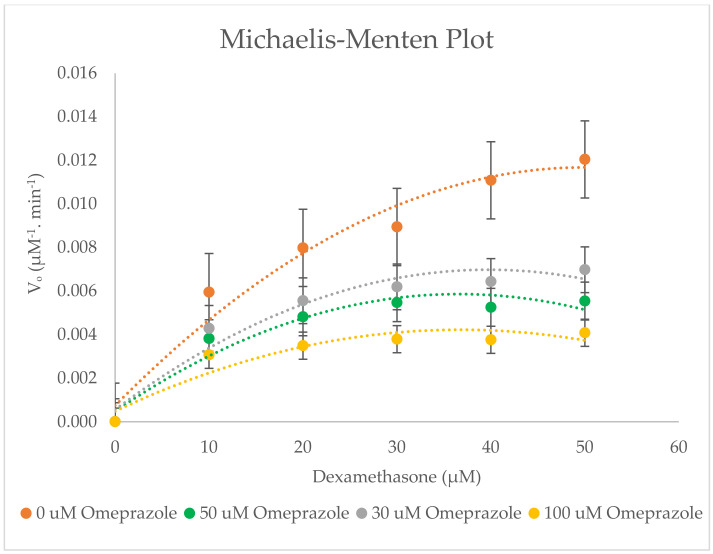
Michaelis−Menten plot for the inhibition of CYP3A2−catalysed dexamethasone 6−hydroxylation by omeprazole (0−100 µM). The polynomial function of order 2 was used to fit the curves. Values are expressed as the average of triplicate measurements.

An enzyme study was carried out to determine the CYP inhibition by omeprazole in RLMs with different concentrations of probe substrates (10–50 µM) in the absence and presence of omeprazole (inhibitor). The data were taken as a mean of triplicate. The Lineweaver–Burk plot and Michaelis–Menten plot (Figure 9 and Figure 10) show the type of inhibition for the selected enzyme activity. The K_m_, V_max_, C_lint_, IC_50_, and K_i_ values (±SD) are determined and summarised in Table 4.

The inhibition of CYP450 enzymes by a number of drugs causes potential clinical consequences [18]. The use of combination drugs cannot be avoided in the treatment for COVID-19. The combination of remdesivir (antiviral drug) and dexamethasone (anti-inflammatory drug) is currently in use to treat the viral infection, as well as reduce the inflammation caused by COVID-19 infection. Evidence from the literature indicates a potential role for the use of corticosteroids and nonsteroidal anti-inflammatory drugs NSAIDs (ibuprofen) in the treatment of COVID-19 patients [19]. Recent literature also confirms that ibuprofen is not associated with worse clinical outcomes in COVID-19 patients [20]. Interestingly, it is reported in a recent study that at therapeutic concentrations, omeprazole improved the anti-SARS-CoV-2 effects of remdesivir [14].

This study provided the first complete in vitro data that enable us to see the interactions of drugs used in COVID-19 treatment and investigate the inhibitory effects of ibuprofen, remdesivir, and omeprazole on CYP3A2 activity, using a High-Performance Liquid Chromatography.

Dexamethasone is metabolised through the CYP3A2 enzyme in rat liver microsomes to 6β-hydroxydexamethasone. The in vitro findings have revealed that CYP3A2 enzyme activity was inhibited non-competitively by ibuprofen in rat liver microsomes at doses between 0 and 200 µM. Based on Table 2, the maximal rate of reaction (V_max_) decreased compared to the V_max_ of uninhibited reaction (negative control assay), while Michaelis constant (K_m_) stayed the same, as the inhibitor is not competing with the substrate for the active site. The ibuprofen concentrations used (50–200 µM) are similar to the amounts found in plasma (49–242 µM), and cause 50% inhibition of CYP3A2 enzyme activity (IC_50_) [21]. As a result, ibuprofen is a weak inhibitor of CYP3A2 isoenzyme activity, with half-maximum inhibitory concentration (IC_50_) = 230.552 ± 2.020 µM and inhibitory constant (K_i_) = 224.981 ± 1.845 µM.

Previous in vitro studies report that the anti-inflammatory agent ibuprofen (COX non-selective inhibitor) has no inhibitory effect on cytokine expression [22]. With IC_50_ of 270 µM, ibuprofen very weakly inhibits the hydrolysis of arachidonoylethanolamide by the enzyme fatty acid amide hydrolase [23]. This result is consistent with our findings. In vivo, peak plasma concentrations (Cmax) of ibuprofen are in the range of 110–150 µM after two single doses of 200 mg from two different ibuprofen preparations [24]. Another in vitro study showed that the short-term administration of ibuprofen in rats did not affect trimethadione metabolism [25]. In our study, ibuprofen showed higher K_i_ and IC_50_ values against dexamethasone, so drug–drug interaction should be unlikely.

It is evident from this in vitro study (Figure 7 and Figure 8), that remdesivir exhibits mixed inhibition (competitive and non-competitive) properties based on graphical inspection of the Lineweaver–Burk plot and remdesivir’s K_i_ value = 22.504 ± 0.008 µM and IC_50_ value 45.007 ± 0.016 µM. The chosen concentration of remdesivir used (30–100 µM) was well above the plasma concentration range of remdesivir (0.1–7.3 µM), after a single dose of 3–225 mg of remdesivir [26]. No inhibition was found at concentrations lower than 30 µM. Table 3 shows that V_max_ and K_m_ are different for each remdesivir concentration, which further confirms the mixed type of inhibition. In a mixed type of inhibition, remdesivir (inhibitor) can bind to the CYP3A2 enzyme at the same time as dexamethasone (substrate) [6]. Though remdesivir binding may influence the CYP3A2 substrate (dexamethasone) binding. It is also possible that remdesivir binds to a different active site of the CYP3A2 enzyme (allosteric effect). Change in the confirmation of CYP3A2 may occur due to the binding of remdesivir to this allosteric site and this has resulted in reducing the substrate affinity for the active site. Furthermore, 50 µM remdesivir concentration can be assumed as saturated concentration because of high K_m_ values as compared to other remdesivir concentrations (30 and 100 µM).

However, a detailed metabolism study (in vitro or in vivo) of remdesivir has not been conducted [26]. Yang reported that remdesivir is a weak inhibitor of CYP3A4, which is consistent with our findings [27]. He also reported that even though remdesivir is a substrate of several CYP isoforms, drug interactions of remdesivir with CYP3A4 inducers or inhibitors were unlikely [27]. Remdesivir’s hepatic clearance is not mediated by metabolic enzymes but is driven by hepatic blood flow.

A compound with an IC_50_ value below 1 µM is considered to be a strong inhibitor, and it is considered to be a weak inhibitor if the IC_50_ value is more than 50 µM [17]. Thus, the high values of IC_50_ of remdesivir in rat liver microsomes would have a low potential of drug interaction and in causing toxicity involving CYP enzymes.

Our in vitro study with omeprazole has shown that it inhibits the CYP3A2 enzyme activity uncompetitively in rat liver microsomes with a K_i_ of 39.175 ± 0.230 µM. The IC_50_ value was twice the value of K_i_, i.e., 78.351 ± 0.460 µM. According to the Lineweaver–Burk plot of enzyme kinetics, a decrease in V_max_ and K_m_ from 0 to 100 µM omeprazole has been observed in the presence of an uncompetitive inhibitor, as presented in Table 4. Interestingly, the omeprazole showed uncompetitive inhibition of CYP3A2 activity. Uncompetitive inhibition is a rare phenomenon for most enzymes. In this case, omeprazole may bind to the enzyme-substrate complex and change (inhibit) the activity of CYP3A2 and, thus, have a very specific effect. Because of the non-productive nature of the ES-inhibitor (ESI) complex, a high concentration of inhibitors can decrease the reaction velocity.

Omeprazole was studied at higher concentrations (30–100 µM) than its therapeutic plasma concentration (1.1–2.0 µM). No inhibition was found at concentrations lower than 30 µM. Keeling et al. reported that in vitro administration of omeprazole for up to 60 min at pH 6.1 or pH 7.4 showed no substantial inhibition of the ATPase activity [28]. This is consistent with our study, as no inhibition of dexamethasone substrate was found. The results from an in vivo study demonstrated that several daily oral doses of omeprazole (40 mg, C_max_ was 1207 ng/mL) had no substantial effect on the pharmacokinetics of roxadustat [29].

Another in vitro study confirmed that omeprazole was a poor inhibitor of bufuralol 1′-hydroxylation with IC_50_ > 200 μM [30]. The high values of IC_50_ and K_i_ of omeprazole in rat liver microsomes would have a low possibility for drug interactions.

This research showing the effects of ibuprofen, remdesivir and omeprazole on dexamethasone metabolism will be useful for further in vivo study of dexamethasone metabolism (CYP3A activity). Additionally, our findings also provide a rationale for the safe and effective administration of these inhibitor drugs with other drugs. However, an interaction potential of dexamethasone with ibuprofen, remdesivir, and omeprazole has to be considered in vivo before a conclusion can be made.

## 3. Materials and Methods

### 3.1. Chemicals

Remdesivir was procured from Tocris Bioscience, UK, and was stored at −20 °C. Dexamethasone from Tokyo Chemical Industry Co., Ltd. (Nihonbashi-honcho, Chuo-ku, Tokyo, Japan) and 4-hydroxyoctanophenone with purity greater than 99% was obtained from Fisher Scientific (Bishop Meadow Road, Loughborough, Leicestershire, UK). Omeprazole was obtained from Carbosynth Ltd. (Axis House, Compton, Berkshire, UK). HPLC grade acetonitrile was purchased from Merck, Co. (Old Brickyard, Gillingham, UK). Ibuprofen was purchased from Sigma-Aldrich, Co. (Spruce Street, St. Louis, MO, USA). Glucose-6-phosphate (G-6-P), phosphoric acid (85% *w*/*w*), EDTA (Ethylenediaminetetraacetic acid), Potassium phosphate monobasic, potassium phosphate dibasic, glucose-6-phosphate dehydrogenase (G-6-PDH), NADP^+^ (Nicotinamide Adenine Dinucleotide Phosphate), and magnesium chloride (MgCl_2_) was purchased from Merck. Ethyl acetate was procured from VWR International Ltd. (Hunter Boulevard, Lutterworth, Leicestershire, UK). 6β-Hydroxydexamethasone was purchased from Cayman Chemical (East Ellsworth Road, Ann Arbor, MI, USA). Diethyl ether was purchased from Fischer Scientific (Bishop Meadow Road, Loughborough, UK).

### 3.2. Rat Liver Microsomes

The pooled liver microsomes from male rats (Sprague Dawley) were purchased from Merck (Old Brickyard Road, Gillingham, UK) and were stored at −80 °C for further analysis.

### 3.3. Instruments

The shaking incubator used for the incubation of the tubes was from Eppendorf UK Limited (Eppendorf House, Arlington Business Park, Whittle Way, Stevenage, UK). A high-performance liquid chromatographic system (LC-2010A HT Shimadzu, Kyoto, Japan) was used for the analysis equipped with a degasser, an autosampler, low-pressure pump quaternary gradient, LC column oven, and a UV detector. The chromatographic data were processed using software LabSolutions. A Waters (Waters Corporation, Milford, MA, USA) C18 column (150 mm × 4.6 mm, 3.5 µm particle size) was used for the analysis.

Assay components (NADPH enzyme, 6β-dexamethasone, dexamethasone, inhibitors (remdesivir, omeprazole, and Ibuprofen) and 4′-hydroxyoctanophenone were separated using an isocratic elution mode. The mobile phase composition was (70% acetonitrile and 30% water, *v*/*v*). The HPLC instrument was controlled at 0.6 mL/min of flow rate, 10 μL of injection volume, the column was set at 25 °C and the detection wavelength was chosen at 243 nm [12]. The results are presented as the standard deviation of triplicate measurements.

### 3.4. Potential Effects of Inhibitors on CYP3A2 Activity In Vitro

To determine the potential effects of ibuprofen, remdesivir and omeprazole on CYP3A2 activity, 6β-hydroxydexamethasone formation after different time intervals was quantified on the HPLC instrument. In microcentrifuge tubes, a final volume of 500 µL contained microsomal protein (0.5 mg/mL), a range of dexamethasone (10, 20, 30, 40 and 50 µM), NADPH (1.0 mM), Glucose-6-Phosphate (5 mM), magnesium chloride (3.0 mM), (Glucose-6-Phosphate Dehydrogenase (1.7 units/mL), 0.067 M potassium phosphate buffer (pH 7.4) and ethylenediaminetetraacetic acid (1.0 mM EDTA). Microcentrifuge tubes containing assay components were incubated at 37 °C for 40 min in the presence of ibuprofen (0, 50, 100 and 200 µM), remdesivir (0, 30, 50 and 100 µM), and omeprazole (0, 30, 50 and 100 µM). The percentage of organic solvent in the assay was not more than 1% *v*/*v*.

Additionally, 15 μM of 4-hydroxyoctanophenone (internal standard) dissolved in ice-cold acetonitrile was added to the reaction tubes to quench the reaction. The quenched reaction masses were transferred to the new vials and substrate and metabolite were double extracted with ethyl acetate (3 mL) and diethyl ether (3 mL), respectively. The organic extracts were evaporated to dryness. The mobile phase (70% acetonitrile and 30% water, *v*/*v*) was used to dissolve the residues. Then, 10 µL of the solution was injected for HPLC analysis.

### 3.5. Analytes Stock and Standard Solutions Preparation

For the cytochrome P3A2 enzyme assay, a stock solution of dexamethasone yielding a 1000 μM concentration was prepared. Serial dilutions of dexamethasone (50, 40, 30, 20, and 10 μM) were prepared from the stock solution in the mobile phase (70% methanol + 30% water, *v*/*v*). The stock solution of 6β-hydroxydexamethasone (2 µM) was prepared in the mobile phase (70% methanol + 30% water, *v*/*v*) and standard solutions (0.2, 0.4, 0.6, 0.8 and 1 µM) were prepared from the stock by serial dilutions. 4-hydroxyoctanophenone powder (0.0010 mg) was dissolved in 10 mL of acetonitrile. The final stock of 15 μM concentration was prepared by combining 165 µL of 4-hydroxyoctanophenone from stock and 49 mL and 835 µL of mobile phase in a volumetric flask.

Remdesivir stock (2 mg/mL) was prepared in a 5 mL volumetric flask in methanol. Serial dilutions of remdesivir (100, 50, and 30 µM) were performed. A stock solution of ibuprofen of 1000 μM was prepared by weighing 1000 mg of ibuprofen and dissolving in 5 mL of methanol. Standard solutions of ibuprofen (50, 100, and 200 μM) were prepared by serial dilution. Omeprazole of 1000 μM was prepared in methanol as a stock solution. Standard solutions of omeprazole (30, 50, and 100 μM) were prepared by serial dilution in the mobile phase (70% methanol + 30% water, *v*/*v*).

### 3.6. Optimization of Substrate Concentration In Vitro

In order to determine the optimal concentrations of dexamethasone (as a probe substrate), a series of dexamethasone concentrations (0, 25, 50, 100, 150 and 200 μM) was added to the incubation system in vitro. The formation of 6β-hydroxydexamethasone (metabolite) was calculated from the standard calibration curve. The optimal concentrations were determined by the linear relationship between the substrate concentrations and metabolite formation.

### 3.7. Statistical Analysis

Statistical analyses were completed using Microsoft Excel 2010 software for kinetic parameters. The concentration of dexamethasone metabolite produced at different time intervals in the presence and absence of different inhibitors (ibuprofen, remdesivir, and omeprazole) were determined from 6β-hydroxydexamethasone calibration curve for CYP3A2 inhibition studies. Analysis of variance (ANOVA) test was also performed (at 0.05 significance level).

In the case of ibuprofen, inhibition data demonstrated non-competitive inhibition. Schwarz criterion (SC) and Akaike information criterion (AIC) were acquired from nonlinear regression analysis. IC_50_ values were calculated by nonlinear regression analysis and GraphPad Prism software was used for this purpose. The following equation was used to calculate the percentage of inhibition, % inhibition = V_max(inh)_ = V_max_/(1 + I/K_i_), in which V_max(inh)_ is the inhibited velocity, Vmax is the maximal rate of reaction, I is inhibitor concentration, and K_i_ is an inhibitory constant.

The type of CYP3A2 inhibition for remdesivir inhibitor was believed to be a mixed type of inhibition based on the Lineweaver–Burk plots shape, AIC, SC, and standard error. The IC_50_ value was calculated by using the formula: V = [V_0_/(1 + (I/IC_50_)^S^)], in which V is the observed velocity, V_0_ is uninhibited velocity, I is the concentration of inhibitor and S is the slope factor.

For omeprazole, inhibition was considered as uncompetitive inhibition. The Lineweaver–Burk plot was used to assess the inhibition parameters such as K_m_, V_max_, and Cl_int_. The following equation was used to calculate these parameters: V = V_max_ × [S]/[S] + K_m_ (1 + [I]/K_i_), in which V_max_ is the maximal rate of the reaction, V is the observed velocity, S is the slope factor, K_i_ is an inhibitory constant, K_m_ is Michaelis–Menten constant, and I is the inhibitor concentration.

## 4. Conclusions

In summary, an in vitro study using rat liver microsomes emphasizes the inhibition of CYP3A2 enzyme activity by ibuprofen, remdesivir, and omeprazole, as these drugs have been used in COVID-19 treatment. Our data demonstrated that ibuprofen, remdesivir, and omeprazole possibly inhibit CYP3A2 enzyme activity as a non-competitive, mixed and uncompetitive inhibition mode, respectively. The outcomes of this research guide the safe use of dexamethasone and other COVID-19 drugs (ibuprofen, remdesivir, and omeprazole) in healthcare screening. More in vivo trials should be performed to assess the safe administration of taking ibuprofen, remdesivir, and omeprazole drugs with dexamethasone for patient care.

## Figures and Tables

**Table 1:** Displaying HPLC investigated parameters.

Investigated Parameters	Characteristic
Mobile phase	70% acetonitrile and 30% H_2_O, *v*/*v*
Column temperature	25 °C
Wavelength	243 nm
Flow rate	0.6 mL/min
Internal standard	4-hydroxyoctanophenone
Injection volume	10 µL
Run time	8 min
Column	Waters C18 column, 15 mm × 4.6 mm, 3.5 µm

**Table 2:** Pharmacokinetic parameters for inhibition patterns of CYP3A2 isoenzyme in the presence of dexamethasone substrates and Ibuprofen (inhibitor). The mean of three experiments is taken ± SD (*n* = 3). Note: *p* < 4.653 × 10^−7^.

Ibuprofen Concentration	Pharmacokinetic Parameters for 6β-Hydroxylase			
	K_m_ (µM)	V_max_ (µM^−1^∙min^−1^)	Cl_int_ (µM^−2^∙min^−1^)	% Inhibition
**0 µM Ibuprofen**	15.967 ± 1.582	0.0151 ± 0.058 × 10^−3^	0.0009 ± 0.093 × 10^−3^	-
**50 µM Ibuprofen**	15.873 ± 1.680	0.0116 ± 0.608 × 10^−3^	0.0007 ± 0.054 × 10^−3^	22.998 ± 4.341
**100 µM Ibuprofen**	15.443 ± 1.034	0.0099 ± 0.040 × 10^−3^	0.0006 ± 0.043 × 10^−3^	34.734 ± 0.336
**200 µM Ibuprofen**	15.153 ± 0.446	0.0092 ± 0.100 × 10^−3^	0.0006 ± 0.024 × 10^−3^	38.939 ± 0.476

V_max_: Maximal reaction rate. K_m_: Michaelis–Menten constant. Cl_int_: Hepatic Intrinsic Clearance.

**Table 3:** V_max_, K_m_ and Cl_int_ values and inhibition patterns of CYP3A2 isoenzyme in the presence of dexamethasone substrates and remdesivir (inhibitor). The mean of three experiments is taken ± SD (*n* = 3). Note: *p* < 0.002.

Remdesivir Concentration	Pharmacokinetic Parameters for 6β-Hydroxylase				
	K_m_ (µM)	V_max_ (µM^−1^∙min^−1^)	Cl_int_ (µM^−2^∙min^−1^)	IC_50_ (µM)	K_i_ (µM)
**0 µM Remdesivir**	9.637 ± 0.550	0.0119 ± 0.700 × 10^−3^	0.0001 ± 0.010 × 10^−3^	45.007 ± 0.016	22.504 ± 0.008
**30 µM Remdesivir**	22.097 ± 0.922	0.0122 ± 0.306 × 10^−3^	0.0006 ± 0.011 × 10^−3^	-	-
**50 µM Remdesivir**	23.167 ± 1.002	0.0146 ± 0.586 × 10^−3^	0.0006 ± 0.018 × 10^−3^	-	-
**100 µM Remdesivir**	13.300 ± 0.436	0.0132 ± 0.153 × 10^−3^	0.0010 ± 0.025 × 10^−3^	-	-

V_max_: Maximal reaction rate. K_m_: Michaelis–Menten constant. Cl_int_: Hepatic Intrinsic Clearance.

**Table 4:** K_m_, V_max_ and Cl_int_ values for 6β-hydroxylase formation. The mean of three experiments is taken ± SD (*n* = 3). Note: *p* < 1.389 × 10 ^−14^.

Omeprazole Concentration	Pharmacokinetic Parameters for 6β-Hydroxylase				
	K_m_ (µM)	V_max_ (µM^−1^∙min^−1^)	Cl_int_ (µM^−2^∙min^−1^)	IC_50_ (µM)	K_i_ (µM)
**0 µM Omeprazole**	16.033 ± 1.498	0.0152 ± 0.208 × 10^−3^	0.0010 ± 0.093 × 10^−3^	78.351 ± 0.460	39.175 ± 0.230
**30 µM Omeprazole**	8.800 ± 1.307	0.0080 ± 0.346 × 10^−3^	0.0009 ± 0.091 × 10^−3^		
**50 µM Omeprazole**	6.423 ± 1.050	0.0062 ± 0.473 × 10^−3^	0.0010 ± 0.093 × 10^−3^		
**100 µM Omeprazole**	3.867 ± 0.230	0.0042 ± 0.058 × 10^−3^	0.0011 ± 0.066 × 10^−3^		

V_max_: Maximal reaction rate. K_m_: Michaelis–Menten constant. Cl_int_: Hepatic Intrinsic Clearance.

## Data Availability

All the data presented in the study is available in this article.

## Sample Availability

Samples of the compounds are available from the authors.
